# DNM1L-mediated fission governs mitophagy & mitochondrial biogenesis during myogenic differentiation

**DOI:** 10.1186/s12964-025-02142-x

**Published:** 2025-04-01

**Authors:** Fasih A. Rahman, Jasmine M. Friedrich Yap, Tyler M. Joseph, Amanda M. Adam, Sarah M. Chapman, Joe Quadrilatero

**Affiliations:** Department of Kinesiology and Health Sciences, Faculty of Health, University of Waterloo, 200 University Ave. West, Waterloo, ON N2L 3G1 Canada

**Keywords:** Mitochondrial fission, Mitophagy, Mitochondrial biogenesis, Apoptosis, Myogenesis, Skeletal muscle

## Abstract

**Background:**

Remodeling of the mitochondrial network is implicated in myogenesis. Remodeling processes including mitochondrial fission, mitophagy, and biogenesis are important as they finetune the mitochondrial network to meet the increased energetic demand of myotubes. Evidence suggests that mitochondrial fission governs other mitochondrial remodeling processes; however, this relationship is unclear in the context of myogenesis.

**Methods:**

We used C2C12 myoblasts to study changes in mitochondrial remodeling processes and their role in regulating myogenesis. To investigate this, we employed genetic manipulation with adenoviruses to modify the levels of key molecules involved in mitochondrial remodeling, including DNM1L, BNIP3, and PPARGC1A.

**Results:**

We demonstrate that overexpression of fission protein DNM1L accelerated mitophagic flux, but reduced myotube size without affecting mitochondrial biogenesis. Conversely, DNM1L knockdown reduced mitophagic flux, impaired myoblast differentiation, and suppressed mitochondrial biogenesis signaling. Additionally, DNM1L knockdown increased mitochondrial apoptotic signaling through CASP9 and CASP3 activation. Attempts to rescue myogenesis through overexpression of the mitophagy receptor BNIP3 or the biogenesis regulator PPARGC1A were unsuccessful in the absence of proper mitochondrial fission. Furthermore, DNM1L overexpression in BNIP3-deficient cells enhanced mitophagic flux, but did not promote myogenesis.

**Conclusion:**

These results underscore the complex interdependencies among mitochondrial remodeling processes and highlight the necessity for sequential activation of mitochondrial fission, mitophagy, and biogenesis.

## Background

Cellular differentiation allows precursor cells to undergo changes in morphology and function to suit a more specialized cell type. For cells to differentiate, they must carefully integrate numerous extracellular and intracellular signals to facilitate the elimination of unspecialized cellular material and repurpose the basic macromolecules to synthesize more specialized cellular material [1]. This process is typically referred to as “remodeling”. One of the major mechanisms of cellular remodeling is autophagy (i.e., self-eating). Autophagy is a degradative process that is used to eliminate damaged proteins, organelles, and other cellular components [1–3]. Autophagy can occur in two forms: bulk or selective. Bulk autophagy involves the non-specific degradation of cellular components and primarily serves as a general response to nutrient deprivation, providing cellular energy and building blocks. In contrast, selective autophagy targets specific organelles or molecules and plays a key role in maintaining cellular homeostasis by removing damaged or dysfunctional components, such as mitochondria (mitophagy) or protein aggregates (aggrephagy). Notably, mitophagy has emerged as a key contributor to differentiation by facilitating metabolic transitions and mitochondrial quality control to support the energy demands and functional requirements of specialized cell types [4, 5].

Various mechanisms of mitophagy have been identified over the last decade. One of the first mechanisms identified is the PTEN-induced kinase 1 (PINK1)-parkin (PRKN) pathway, which is involved in the removal of damaged/depolarized mitochondria [6]. Alternatively, mitophagy receptors have been identified, and are implicated in the removal of mitochondria during cellular differentiation [4, 7, 8]. The removal of mitochondria is particularly relevant in specialized cells that have a high metabolic demand, such as skeletal muscle cells (i.e., myoblasts). Recent evidence from our lab [4, 9], and others [5] demonstrate that a lack of autophagy or mitophagy severely inhibits myogenic differentiation. Furthermore, the lack of degradation of “immature” mitochondria is an important mechanism preventing the rebuilding of the mitochondrial network needed for myogenic differentiation [4, 5].

The role of mitophagy is apparent during myogenic differentiation; however, emerging evidence suggests that the state of the mitochondrial network may be a regulatory mechanism for mitophagy. For instance, hyperfusion of mitochondria severely halts mitophagy, whereas the fragmentation of mitochondria plays an important preceding step for mitophagy [10]. Additionally, the fragmentation of mitochondria results in smaller, less functional mitochondria that exhibit lower membrane potential, which is a major signal for mitophagy [10]. Conversely, hyperfusion of mitochondria presents physical limitations for autophagic machinery; therefore limiting mitophagic flux [10]. Nevertheless, the regulation of the mitochondrial network and its regulatory role in mitophagy is only starting to be unraveled. Previous work from our lab has demonstrated that the inhibition of fission molecule dynamin 1 like (DNM1L) significantly impairs myogenic differentiation [11]; however, it is unclear whether this is a result of reduced mitophagy. Moreover, the regulatory role of DNM1L on other mitochondrial remodeling processes such as mitochondrial biogenesis remains elusive.

Therefore, the purpose of the present study was to determine the regulatory role of DNM1L on mitochondrial remodeling processes during myogenic differentiation. We further questioned whether enhanced mitophagy or mitochondrial biogenesis can restore myogenic differentiation in a fission-deficient state. We also questioned whether increased mitochondrial fission could facilitate differentiation independent of BCL2-interacting protein 3 (BNIP3)-mediated mitophagy.

## Material and methods

### Cell culture

C2C12 murine myoblasts were cultured as previously described [4, 9, 11]. Cells were maintained in growth media consisting of low-glucose Dulbecco’s Modified Eagles Medium (DMEM) supplemented with 5% fetal bovine serum, 5% Serum Plus II, and 1% penicillin/streptomycin on polystyrene culture dishes. Differentiation was induced at 80% confluency with the addition of differentiation medium consisting of low-glucose DMEM supplemented with 2% horse serum and 1% penicillin/streptomycin. Cells were collected at Day 0 (D0; proliferating) or Days 1-5 (D1, D2, D3, D5; differentiating) by trypsinization, centrifugation at 1000 × g, and stored at −80 °C until analyses.

Knockout of *Bnip3* in C2C12 cells was achieved with CRISPR/Cas9 vectors targeting mouse *Bnip3*. Guide RNA (gRNA) targets were identified using the following tools: Zhang Lab, MIT (https://zlab.bio/guide-design-resources), CCTop (https://cctop.cos.uni-heidelberg.de:8043/), and Off-Spotter (https://cm.jefferson.edu/Off-Spotter/). Two gRNA sequences were identified: First: 5ʹGAGCCACCATGTCGCAGAGC(GGG), and 5ʹGGAGGAGAACCTGCAGGGTG(AGG). Corresponding oligonucleotides were constructed (Sigma) to allow cloning into the CRISPR/Cas9 vector pSpCas9(BB)−2A-puro (PX459) V2.0 (Addgene, 62988), which uses a single gRNA. Cloning was confirmed by sequencing constructs (Center for Applied Genomics, Sick Kids Hospital, Toronto, Canada).

### Adenovirus amplification and precipitation

Adenoviruses were amplified using HEK-293 cells as previously described [4]. Briefly, HEK-293 cells were grown until 60% confluency. Growth medium was refreshed, and the following adenoviruses were added at an MOI of 5: Ad-*Gfp,* Ad-*Gfp-Ppargc1a* (kindly provided by Dr. Beatrice Filippi, University of Leeds), Ad-*LacZ*, Ad-sh*Dnm1l*, Ad-*Dnm1l* (kindly provided by Dr. Junichi Sadoshima, Rutgers University), Ad-*BNIP3* (kindly provided by Dr. Abhinav Diwan, Washington University), and Ad-CMV-*Cox8*-*EGFP*-*mCherry* (kindly provided by Dr. Wen-Xing Ding, University of Kansas). Cells were incubated until 90% cytopathic effect was observed and cells detached from the plate. The cell suspension was collected, centrifuged at 1000 × g for 15 min, and the supernatant was transferred to a fresh tube. The remaining cell pellet was resuspended in a small volume of PBS, freeze/thawed three times, and centrifuged at 3000 × g for 15 min to pellet cellular debris. The resulting supernatant was pooled into the previously collected supernatant. Adenoviruses were precipitated using polyethylene glycol/NaCl (10% PEG-8000/0.625 M NaCl final concentration) or protamine sulfate-chondroitin sulfate C (100 μg/ml each) overnight on a rocker. The following day, tubes were centrifuged at 3000 × g for 30 min to pellet the adenovirus and the supernatant was discarded. All viruses were stored in PEG-PBS at −80 °C with no loss in viral titers after multiple freeze/thaw cycles.

### Adenoviral transduction of myoblasts

All adenovirus experiments were performed in 24-well plates. C2C12 myoblasts were plated at 2 × 10^4^ per well. On the subsequent day, cells were washed in PBS and adenoviruses were added at an MOI of 30–100 in serum-free DMEM supplemented with 8 μg/ml polybrene for 1–2 h at 37 °C. Following incubation, cells were washed in PBS to remove residual virus, and growth media was added to allow cells to recover from viral load. Cells were differentiated on the following day as described earlier.

### Immunoblotting

Immunoblotting was performed as previously described [4, 9, 11, 12]. Pelleted cells were incubated in ice-cold lysis buffer (20 mM HEPES, 10 mM NaCl, 1.5 mM MgCl_2_, 1 mM DTT, 20% glycerol, 0.1% Triton-X100, pH 7.4) containing protease inhibitor and sonicated at 40 Hz for 10 s. Protein content was determined using a BCA protein assay. Equal protein was loaded and separated using 8–12% SDS-PAGE, transferred on to PVDF membranes, and blocked with 2–5% non-fat milk powder in TBS-T at room temperature for 1 h. Membranes were briefly rinsed and incubated overnight in primary antibodies against: BNIP3 (#3769, Cell Signaling), BNIP3L (#12396, Cell Signaling), MAP1LC3B/LC3B (#2775, Cell Signaling), GAPDH (#2118, Cell Signaling), OPA1 (#80471, Cell Signaling), MFN2 (#9482, Cell Signaling), DNM1L (#8570, Cell Signaling), SQSTM1 (PM045, MBL), PINK1 (sc-33796, Santa Cruz), VDAC1 (sc-390996, Santa Cruz), ANT1 (sc-9299, Santa Cruz), CYCS (sc-13256, Santa Cruz), PPARGC1A (ST1202, Sigma), SOD1 (SOD-101, Stressgen), SOD2 (SOD-110, Stressgen), myosin heavy chain (MYH; MF-20, DSHB), and myogenin (MYOG; F5D, DSHB). Following incubation, membranes were washed, and appropriate horseradish peroxidase-conjugated secondary antibody was applied at room temperature for 1 h. Bands were visualized using ECL substrate on a ChemiDoc Imaging System (BioRad).

### Immunocytochemistry

MYH was stained to determine differentiation capacity of myoblasts. Briefly, cells were differentiated for 5-days (D5), fixed in 4% formaldehyde, and blocked in 5% goat serum in PBS containing 0.1% Triton-X100 (PBS-T) at room temperature. MYH antibody (1:400; MF-20, DSHB) was applied for 2 h at room temperature. Cells were washed with PBS-T prior to adding the appropriate Alexa Fluor secondary antibody and counterstaining with 300 nM DAPI for 1 h at room temperature. Cells were washed several times and 0.5 ml of PBS added to each well prior to imaging. All images were collected using the Cytation 5 Imaging Multi-Mode Reader at 20X magnification.

### Flow cytometry

Myoblasts were cultured on 24-well plates, treated with appropriate adenoviruses, and grown until confluent. Cells were differentiated for 24-h (i.e., D1), collected, centrifuged, washed in PBS, and resuspended in PBS containing 1 g/L glucose with 50 nM TMRE and 50 nM MitoTracker Green (MTG) to determine mitochondrial membrane potential and mitochondrial content, respectively. Mitochondrial permeability transition pore (MPTP) formation was assessed by resuspending cells in PBS with 1 g/L glucose containing 1 μM Calcein AM and 1 mM CoCl_2_. CoCl_2_ quenches Calcein fluorescence and is permeable to the plasma membrane but not the inner mitochondrial membrane [13]. Therefore, healthy cells co-loaded with Calcein-AM and CoCl_2_ results in quenching of cytosolic Calcein florescence without affecting mitochondrial fluorescence. In contrast, MPTP opening facilitates permeability of CoCl_2_, allowing it to pass the IMM and enter the mitochondrial matrix to quench Calcein fluorescence (i.e., lower fluorescence). All samples were incubated at 37 °C with gentle rocking for 30 min before being diluted in PBS with 2% BSA and analyzed using a BD FACSCalibur flow cytometer equipped with Cell Quest Pro software (BD Bioscience).

### Confocal microscopy

C2C12 myoblasts were grown on coverslips coated with Cultrex (Trevigen, 3432–010-01) in a 24-well plate and transduced in the same manner with the addition of Ad-CMV-*Cox8*-*EGFP*-*mCherry*. The growth media was refreshed, and other adenoviruses were added as previously indicated. Cells were grown until 80% confluency, differentiated, and fixed in 4% formaldehyde. Coverslips containing cells were briefly washed in PBS and mounted on microscope slides using Fluoromount-G (Thermo Fisher Scientific) mounting medium. Ten to thirty cells were imaged on a Zeiss LSM 800 confocal microscope at 63X magnification. Mitophagic area was determined using the threshold function on ImageJ.

### Mitochondrial network analysis

Confocal microscopy images of cells transduced with Ad-CMV-*Cox8*-*EGFP*-*mCherry* were exported and subsequently analyzed using a custom macro developed for ImageJ, as previously discussed [14]. First, merged channel images were converted to 8-bit grayscale format to standardize intensity values across all samples. These images underwent a deconvolution process using the Iterative Deconvolution 3D plugin, which applies a theoretical point spread function over ten iterations to correct for inherent optical distortion. To enhance the detectability of network features, the contrast of these images was increased. Following contrast enhancement, images were processed through a Gaussian Blur filter to reduce noise, enhancing feature extraction during subsequent analysis steps. The images were then converted into binary masks to isolate and define structural elements within the mitochondrial network. The refined images were analyzed using the Skeleton 2D/3D plugin to quantitatively assess network morphology. Quantitative data extracted from this analysis were compiled and saved into a spreadsheet format. A custom Python script was used to synthesize data from multiple spreadsheets.

### Caspase and calpain activity assay

To measure caspase (CASP) and calpain (CAPN) activity, whole‐cell lysates were collected at D1 as described above in the absence of protease inhibitors. Samples were incubated in duplicates with 20 μM of Ac‐DEVD‐AFC (AAT Bioquest, 13,401) for CASP3 or Ac‐LEHD‐AFC (Tocris Bioscience, 1575) for CASP9 in assay buffer (20 mM HEPES pH 7.4, 10 mM DTT, and 10% glycerol). For CAPN activity, samples were incubated in duplicates with 20 μM of Suc-LLVY-AMC (Enzo Life Sciences, BML-P802) and Z-LL-CHO (Enzo Life Sciences, BML-PI116) inhibitor in assay buffer for 2 h at 30 °C. Fluorescence measurements were performed at room temperature using a Cytation 5 Imaging Multi‐Mode Reader. The AFC probe was measured with excitation and emission wavelengths at 400 and 505 nm, respectively. The AMC probe was measured with excitation and emission wavelengths at 360 and 440 nm, respectively. The fluorescence values were normalized to protein concentration of samples and expressed as fold changes in fluorescence.

### Statistics

Statistical analyses were performed in GraphPad PRISM as previously described [4, 9, 11]. T-tests were performed to assess differences in treatment within a given time-point. A *p*-value less than 0.05 (i.e., *p* < 0.05) was considered statistically significant, whereas a *p*-value between 0.05 and 0.10 (i.e., *p* ≤ 0.10) was considered a statistical trend for all analyses.

## Results

### Overexpression of DNM1L promoted mitochondrial fragmentation, increased mitophagic flux, and reduced myotube size

Dynamin 1 like protein (DNM1L) is a key regulator of mitochondrial fission which has also been shown to play a role in myogenesis [11, 15]. We aimed to investigate the role of DNM1L and its impact on other mitochondrial remodeling processes during myogenesis. To fully understand the role of DNM1L during myogenesis, we first questioned whether the targeted genetic overexpression (OE) of DNM1L would alter the mitochondrial network during myogenesis. To address this question, we transduced cells with Ad-*Dnm1l* or control virus (Ad-*LacZ*) and examined several mitochondrial and myogenic parameters. Furthermore, we also transduced cells with Ad-CMV-*Cox8*-*EGFP*-*mCherry*, a dual-fluorescence reporter virus used to accurately measure mitophagic flux.

**Fig. 1:**
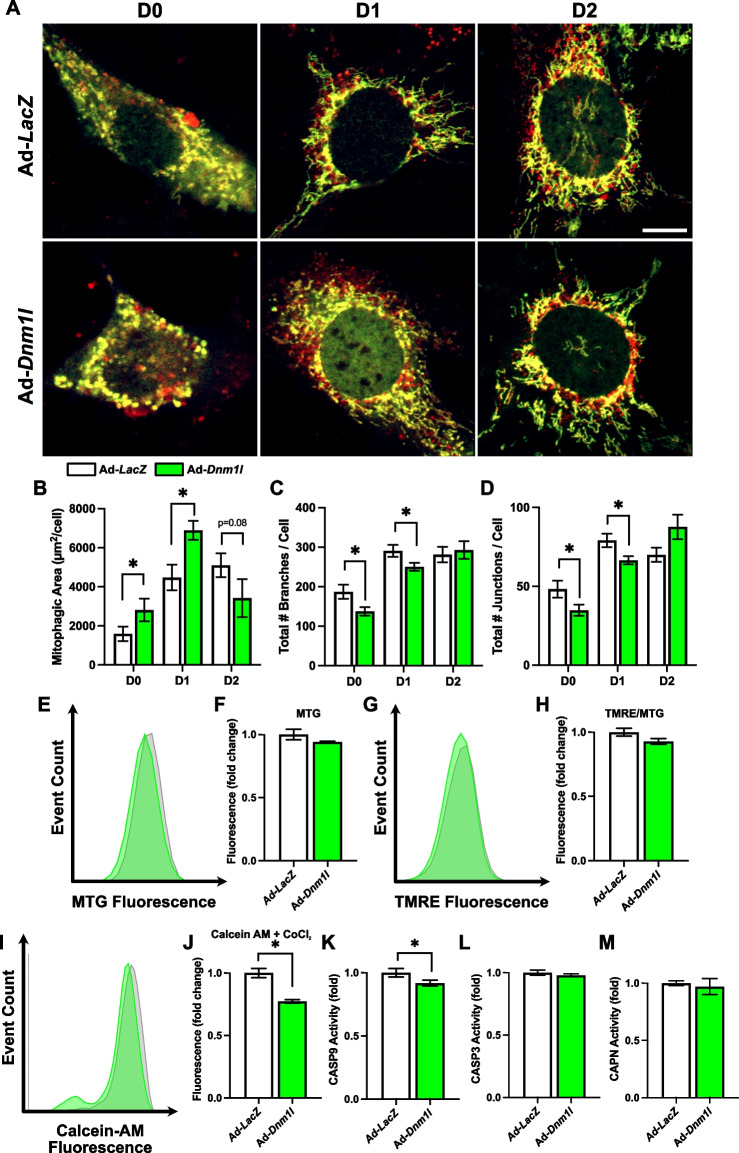
DNM1L OE induces mitochondrial fragmentation and upregulation of mitophagy during myogenesis. **A** Representative images of myoblasts transduced with Ad-CMV-*Cox8*-*EGFP*-*mCherry* to detect mitophagic flux. Mitochondria are dual‐labeled EGFP and mCherry (i.e., yellow). EGFP florescence is reduced during mitochondria degradation resulting in red fluorescence only (i.e., red puncta) and is indicative of mitophagic flux. Scale bar indicates 10 μm. Quantification of (**B**) mitophagic area, (**C**) total mitochondrial branch count, and (**D**) total mitochondrial junction count per cell. Representative histograms and quantification of (**E–F**) MitoTracker Green (MTG) fluorescence, (**G-H**) TMRE fluorescence, and (**I-J**) Calcein-AM + CoCl_2_ fluorescence. Quantification of (**K**) CASP9, (**L**) CASP3, and (**M**) CAPN activity at D1. * *p* < 0.05 compared to Ad-*LacZ* group within the same time point. *n* = 4–30 per group

DNM1L OE resulted in marked mitochondrial fragmentation, as shown by reduced total mitochondrial network branch number (−27% at D0 and −14% at D1; *p* < 0.05) and junction number (−28% at D0 and −18% at D1; *p* < 0.05; Fig. 1A-D), which was accompanied by an early elevation in mitophagic flux (+ 75% at D0 and + 58% at D1; *p* < 0.05). The mitochondrial network appeared to normalize by D2, accompanied by a non-significant reduction in mitophagic flux (−30%; *p* = 0.08; Fig. 1B-D). This temporal pattern suggests a transient impact of DNM1L OE on mitochondrial dynamics and mitophagy. DNM1L OE did not alter MitoTracker green (i.e., mitochondrial mass; MTG) or normalized tetramethylrhodamine ethyl ester (i.e., mitochondrial membrane potential; TMRE) fluorescence but reduced mitochondrial retention of Calcein-AM fluorescence (−23%; *p* < 0.05) suggesting the induction of mitochondrial permeability transition pore (MPTP) opening at D1 (Fig. 1E-J). Interestingly, this was accompanied by reduced caspase 9 (CASP9; −9%; *p* < 0.05) activity, without affecting caspase 3 (CASP3) or calpain (CAPN) activity at D1 (Fig. 1K-M). This shows that DNM1L-mediated fission also influences mitochondrial apoptotic signaling, perhaps independent of MPTP opening.

**Fig. 2:**
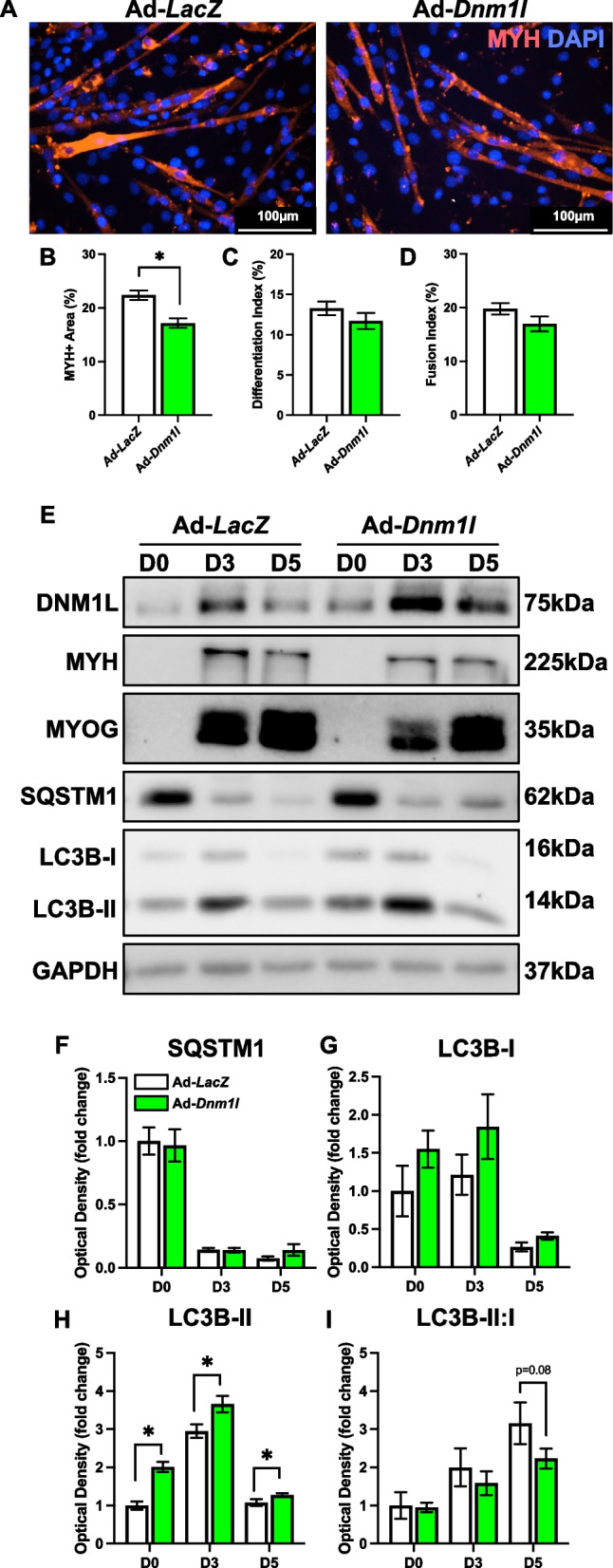
DNM1L OE does not modify myogenesis but reduced myotube size. **A** Representative MYH (red) and DAPI (blue) stain of D5 myotubes. Scale bar indicates 100 μm. Quantification of (**B**) MYH + area, **C**) differentiation index, and (**D**) fusion index. Representative immunoblots (**E**) and quantification of (**F**) SQSTM1, (**G**) LC3B-I, (**H**) LC3B-II, and (**I**) LC3B-II:I. * *p* < 0.05 compared to Ad-*LacZ* group within the same time point. *n* = 4–12 per group

**Fig. 3:**
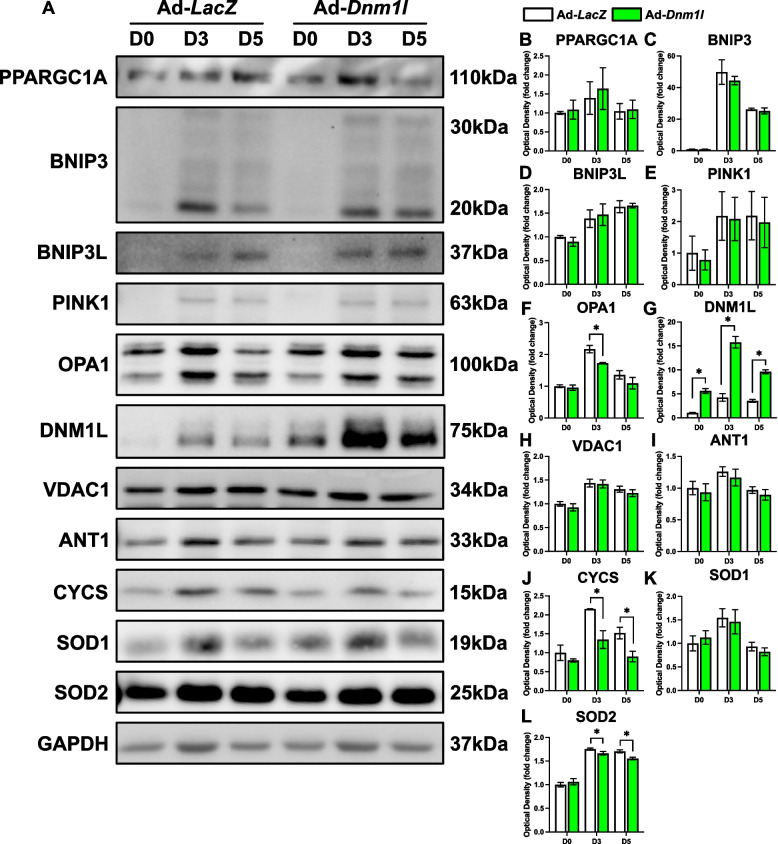
Molecular changes in mitochondrial remodeling proteins in response to DNM1L OE. Representative immunoblots (**A**) and quantification of (**B**) PPARGC1A, (**C**) BNIP3, (**D**) BNIP3L, (**E**) PINK1, (**F**) OPA1, (**G**) DNM1L, (**H**) VDAC1, (**I**) ANT1, (**J**) CYCS, (**K**) SOD1, and (**L**) SOD2. * *p* < 0.05 compared to Ad-*LacZ* group within the same time point. *n* = 4 per group

DNM1L OE led to a significant reduction in myotube size at D5 (−23%; *p* < 0.05) without affecting the differentiation or fusion indices (Fig. 2A-D). This suggests that while DNM1L plays a critical role in myotube formation, its overexpression primarily impacts cell size rather than the differentiation process itself. Additionally, we observed no alteration in SQSTM1 but early elevations in LC3B-II (+ 100% at D0 and + 24% at D3; *p* < 0.05), likely signaling an initial increase in autophagic activity (Fig. 2E-H). Although there was a decline in the LC3B-II:I ratio by D5, this change was not statistically significant (*p* = 0.08) (Fig. 2I). Despite these changes, there were no alterations in proteins related to mitochondrial biogenesis (i.e., PPARG coactivating 1 alpha [PPARGC1A]) or mitophagy (i.e., BNIP3, BNIP3 like [BNIP3L], and PINK1) (Fig. 3A-E). However, DNM1L OE was associated with reductions in key mitochondrial proteins including cytochrome c (CYCS), and superoxide dismutase 2 (SOD2) at D3 and D5 (*p* < 0.05; Fig. 3A, J and A, L), perhaps indicating impairments in mitochondrial electron transport efficiency and antioxidant capacity in the newly formed myotubes.

### Loss of DNM1L impaired mitophagic flux, limited myogenesis, and increased mitochondrial apoptotic signaling

**Fig. 4:**
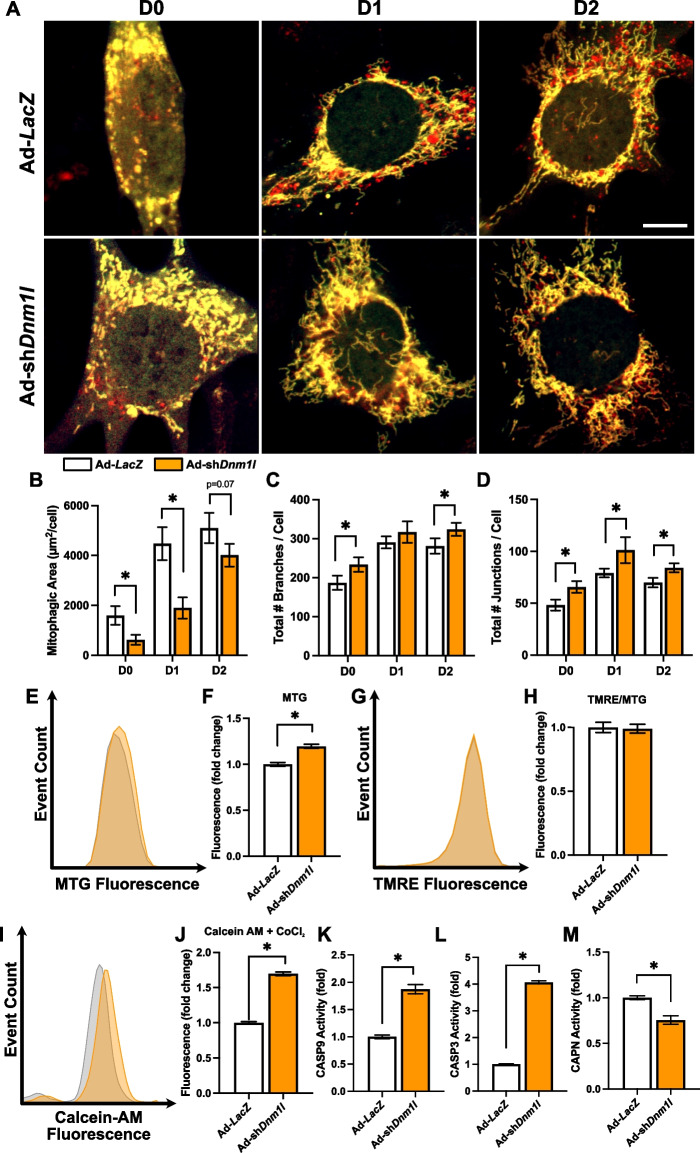
DNM1L KD suppressed mitochondrial fission and mitophagy during myogenesis. **A** Representative images of myoblasts transduced with Ad‐CMV‐*Cox8*‐*EGFP*‐*mCherry* to detect mitophagic flux. Mitochondria are dual‐labeled EGFP and mCherry (i.e., yellow). EGFP florescence is reduced during mitochondria degradation resulting in red fluorescence only (i.e., red puncta) and is indicative of mitophagic flux. Scale bar indicates 10 μm. Quantification of (**B**) mitophagic area, (**C**) total mitochondrial branch count, and (**D**) total mitochondrial junction count per cell. Representative histograms and quantification of (**E–F**) MitoTracker Green (MTG) fluorescence, (**G-H**) TMRE fluorescence, and (**I-J**) Calcein-AM + CoCl_2_ fluorescence. Quantification of (**K**) CASP9, (**L**) CASP3, and (**M**) CAPN activity at D1. * *p* < 0.05 compared to Ad-*LacZ* group within the same time point. *n* = 4–30 per group

Previous work has utilized pharmacological and genetic approaches to limit DNM1L-mediated mitochondrial fission via treatment of cells with mdivi-1 or dominant negative DNM1L^K38A^ [11, 15]. To further understand the regulatory role of DNM1L on mitochondrial remodeling during myogenesis, we used an adenovirus approach to knockdown (KD) DNM1L. DNM1L KD increased mitochondrial branch number (+ 25% at D0 and + 15% at D2; *p* < 0.05; Fig. 4C) and junction number (+ 36% at D0, + 26% at D1, and + 21% at D2; *p* < 0.05; Fig. 4D), which was accompanied by suppressed mitophagic flux at D0 (−63%; *p* < 0.05) and D1 (−57%; *p* < 0.05; Fig. 4A-B). Moreover, DNM1L KD increased MTG fluorescence (+ 19%; *p* < 0.05) but there was no alteration in normalized TMRE fluorescence (Fig. 4E-H). These data suggest that DNM1L KD alters mitochondrial dynamics, shifting the network towards a hyperfused morphology. Furthermore, the hyperfused morphology appears to limit mitochondrial degradation without significantly affecting mitochondrial membrane potential. Interestingly, DNM1L KD increased Calcein-AM retention in mitochondria (+ 69%; *p* < 0.05), suggestive of reduced MPTP opening (Fig. 4I-J). We also observed reduced CAPN activity (−25%; *p* < 0.05), with significantly elevated CASP9 (+ 87%; *p* < 0.05) and CASP3 (fourfold increase; *p* < 0.05) activity at D1 in DNM1L KD cells (Fig. 4K-M). These findings indicate that DNM1L KD affects apoptotic pathways, shifting the balance towards increased mitochondrial CASP activation due to failed mitochondrial fragmentation and subsequent reduced mitophagic flux.

**Fig. 5:**
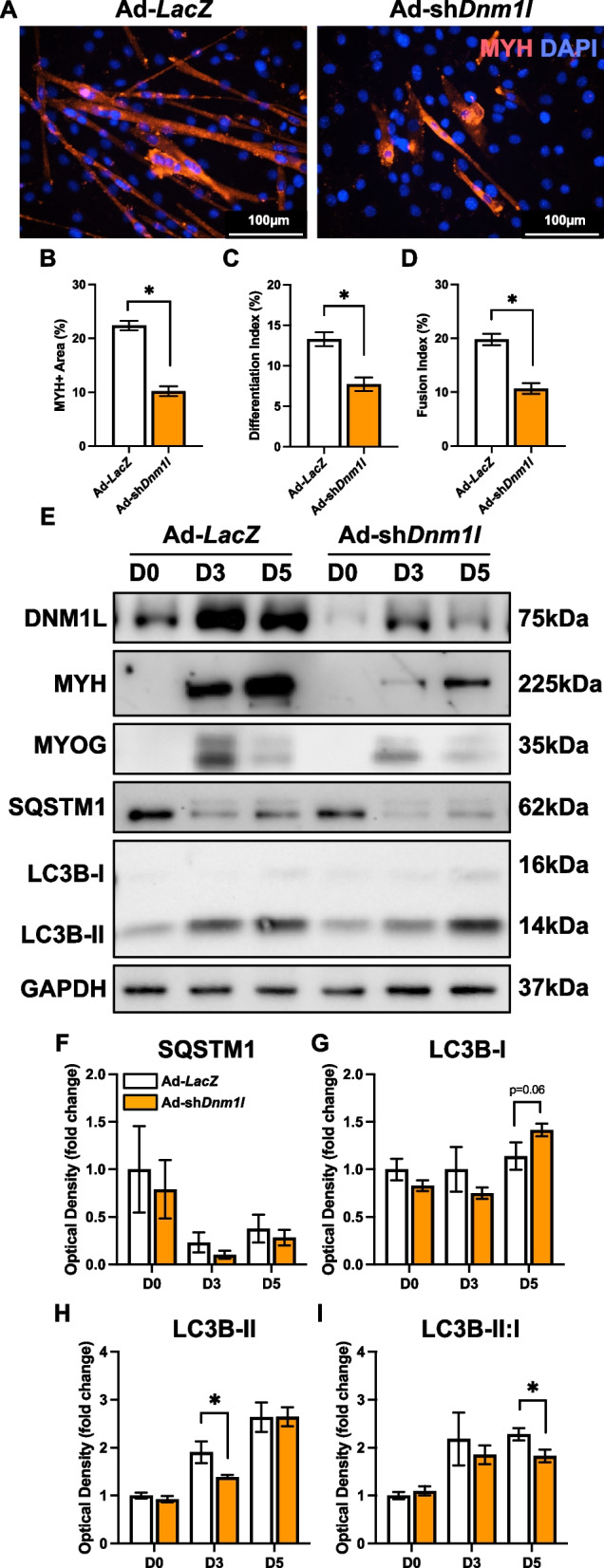
DNM1L KD impaired myogenic differentiation. **A**)Representative MYH (red) and DAPI (blue) stain of D5 myotubes. Scale bar indicates 100 μm. Quantification of (**B**) MYH + area, (**C**) differentiation index, and (**D**) fusion index. Representative immunoblots (**E**) and quantification of (**F**) SQSTM1, (**G**) LC3B-I, (**H**) LC3B-II, and (**I**) LC3B-II:I. * *p* < 0.05 compared to Ad-*LacZ* group within the same time point. *n* = 4–12 per group

**Fig. 6:**
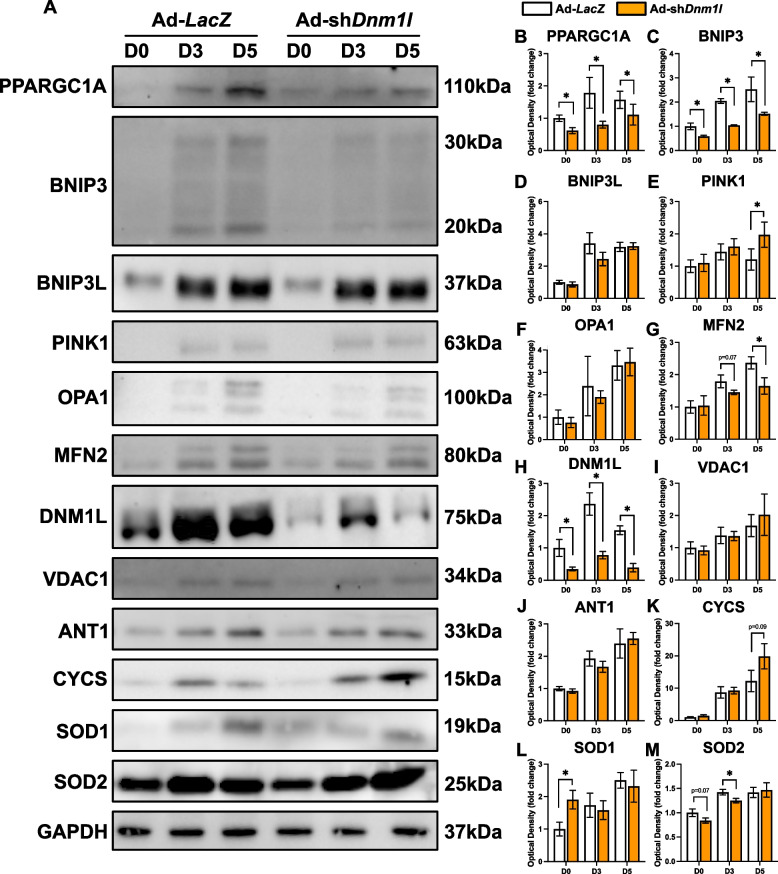
Molecular changes in mitochondrial remodeling proteins in response to DNM1L KD. Representative immunoblots (**A**) and quantification of (**B**) PPARGC1A, (**C**) BNIP3, (**D**) BNIP3L, (**E**) PINK1, (**F**) OPA1, (**G**) MFN2, (**H**) DNM1L, (**I**) VDAC1, (**J**) ANT1, (**K**) CYCS, (**L**) SOD1, and (**M**) SOD2. * *p* < 0.05 compared to Ad-*LacZ* group within the same time point. *n* = 4 per group

Similar to previous findings [11, 15], we showed that DNM1L KD significantly reduced myotube size (−55%; *p* < 0.05), differentiation (−40%; *p* < 0.05) and fusion (−45%; *p* < 0.05) indices at D5 (Fig. 5A-D). Accompanying this reduction, we observed depressed LC3B-II at D3 (−28%; *p* < 0.05) and a decline in the LC3B-II:I ratio at D5 (−36%; *p* < 0.05) in DNM1L KD cells, perhaps hinting at impaired autophagosome formation (Fig. 5E-I). This indicates that DNM1L is crucial for proper myotube formation, and its reduction disrupts the formation and growth of myotubes. Additionally, DNM1L KD led to approximately a 50% reduction in key remodeling molecules including PPARGC1A and BNIP3 throughout the differentiation time course (*p* < 0.05; Fig. 6A-C), although PINK1 increased at D5 (+ 64%; *p* < 0.05; Fig. 6A, E). We also observed other modifications in signaling including reduced MFN2 at D5 (−31%; *p* < 0.05; Fig. 6A, G), reduced SOD2 at D3 (−13%; *p* < 0.05; Fig. 6A, M), and increased SOD1 at D0 (+ 90%; *p* < 0.05; Fig. 6A, L) in DNM1L KD cells. Collectively, these data underscore the complex role of DNM1L in regulating myotube size, myoblast differentiation, mitochondrial dynamics, and apoptosis.

### Overexpression of BNIP3 promoted mitophagic flux and suppressed mitochondrial apoptotic signaling in fission-deficient myoblasts during differentiation

**Fig. 7:**
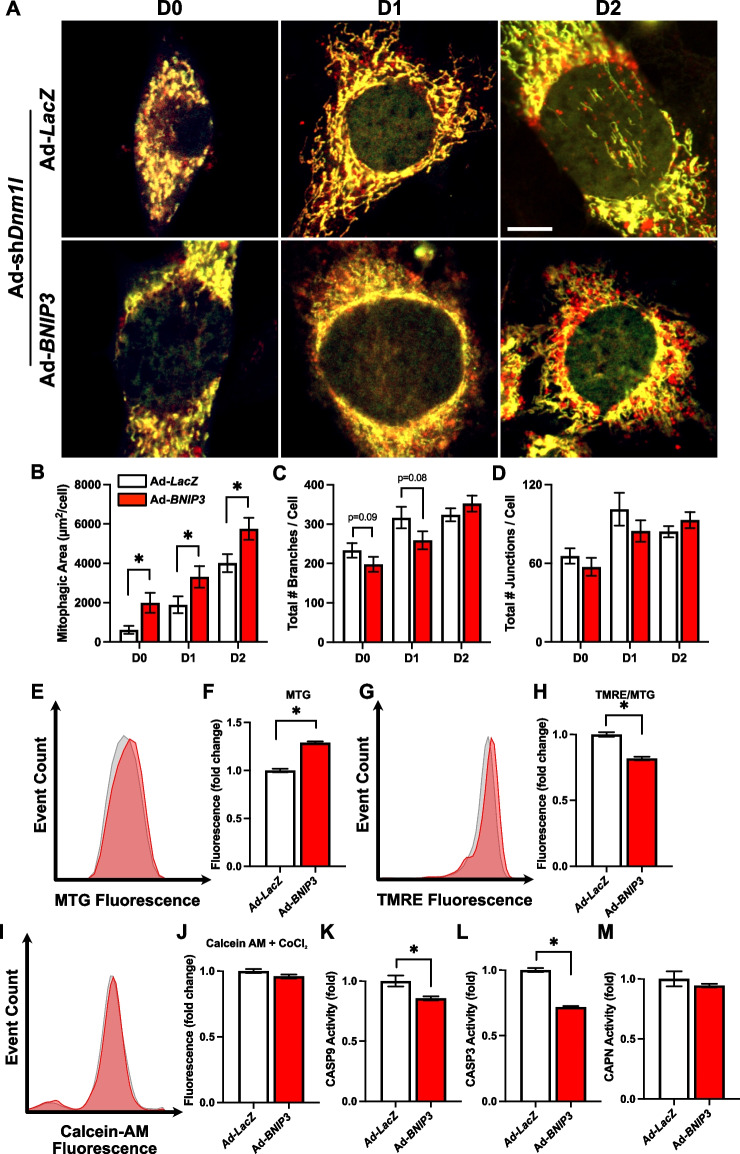
BNIP3 OE activates mitophagy and suppressed CASP activity in fission-deficient cells. **A** Representative images of myoblasts transduced with Ad-CMV-*Cox8*-*EGFP*-*mCherry* to detect mitophagic flux. Mitochondria are dual‐labeled EGFP and mCherry (i.e., yellow). EGFP fluorescence is reduced during mitochondria degradation resulting in red fluorescence only (i.e., red puncta) and is indicative of mitophagic flux. Scale bar indicates 10 μm. Quantification of (**B**) mitophagic area, (**C**) total mitochondrial branch count, and (**D**) total mitochondrial junction count per cell. Representative histograms and quantification of (**E–F**) MitoTracker Green (MTG) fluorescence, (**G-H**) TMRE fluorescence, and (**I-J**) Calcein-AM + CoCl_2_ fluorescence. Quantification of (**K**) CASP9, (**L**) CASP3, and (**M**) CAPN activity at D1. * *p* < 0.05 compared to Ad-*LacZ* group within the same time point. *n* = 4–30 per group

Given our findings that DNM1L is critical in regulating mitophagy and myogenesis, we questioned whether enhanced mitophagy through overexpression of the critical mitophagy receptor BNIP3, which we have previously shown to be important in myogenesis [4], would enhance mitophagy and modify the myogenic response to fission-deficiency. Despite the known dependence of BNIP3-induced mitophagy on fission [16], BNIP3 OE was sufficient to promote mitophagy in DNM1L KD cells (+ 320% at D0, + 75% at D1, and + 42% at D2; *p* < 0.05; Fig. 7A-B). Additionally, BNIP3 OE led to a slight reduction in the number of mitochondrial branches per cell at D0 (*p* = 0.09) and D1 (*p* = 0.08), hinting at the potential role of BNIP3 on mitochondrial fragmentation independent of DNM1L (Fig. 7C). This slight morphological change was accompanied by increased MTG fluorescence (+ 29%; *p* < 0.05; Fig. 7E-F), suggesting greater mitochondrial mass, while the normalized TMRE fluorescence was reduced at D1 (−19%; *p* < 0.05; Fig. 7G-H). Furthermore, BNIP3 OE in fission-deficient cells reduced CASP3 (−29%; *p* < 0.05) and CASP9 (−15%; *p* < 0.05) activity, without affecting mitochondrial Calcein-AM retention (i.e., MPTP opening) or CAPN activity at D1 (Fig. 7I-M). Collectively, these findings indicate that the upregulation of mitophagy, mediated by BNIP3 OE, may attenuate apoptotic pathways by facilitating the degradation of dysfunctional mitochondria.

**Fig. 8:**
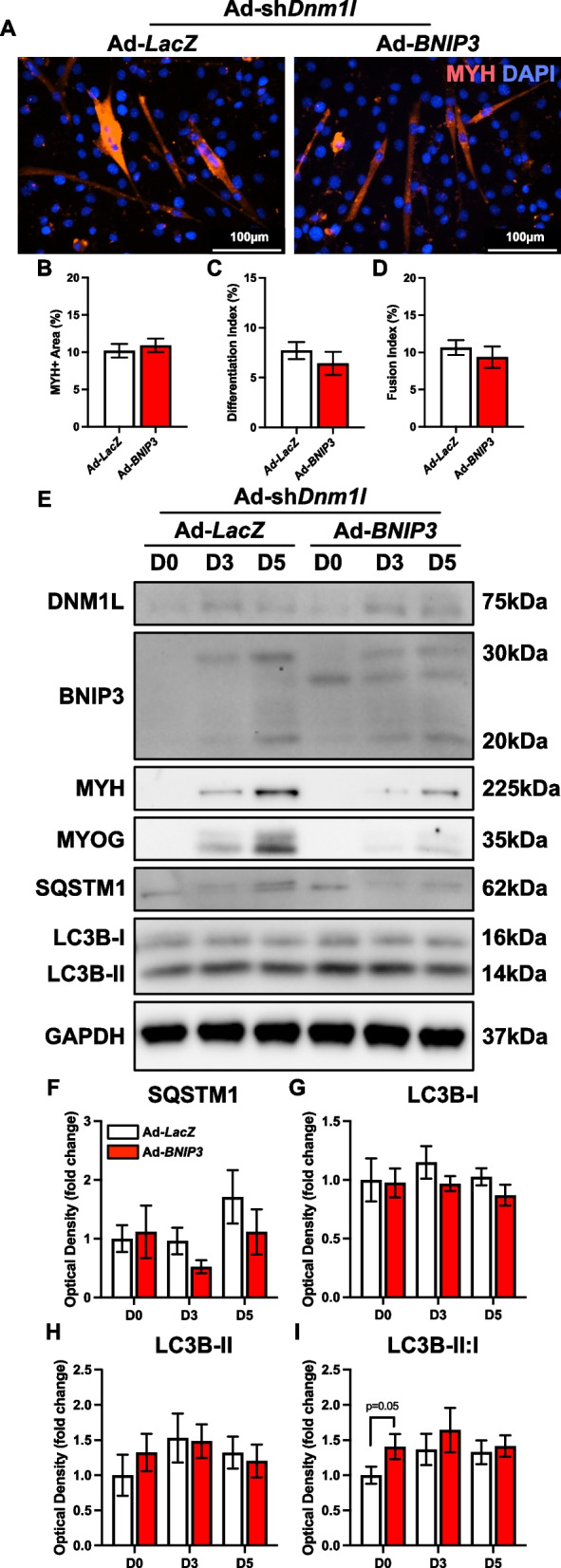
BNIP3 OE in fission-deficient cells does not modify myogenic differentiation. **A** Representative MYH (red) and DAPI (blue) stain of D5 myotubes. Scale bar indicates 100 μm. Quantification of (**B**) MYH + area, (**C**) differentiation index, and (**D**) fusion index. Representative immunoblots (**E**) and quantification of (**F**) SQSTM1, (**G**) LC3B-I, (**H**) LC3B-II, and (**I**) LC3B-II:I. * *p* < 0.05 compared to Ad-*LacZ* group within the same time point. *n* = 4–9 per group

**Fig. 9:**
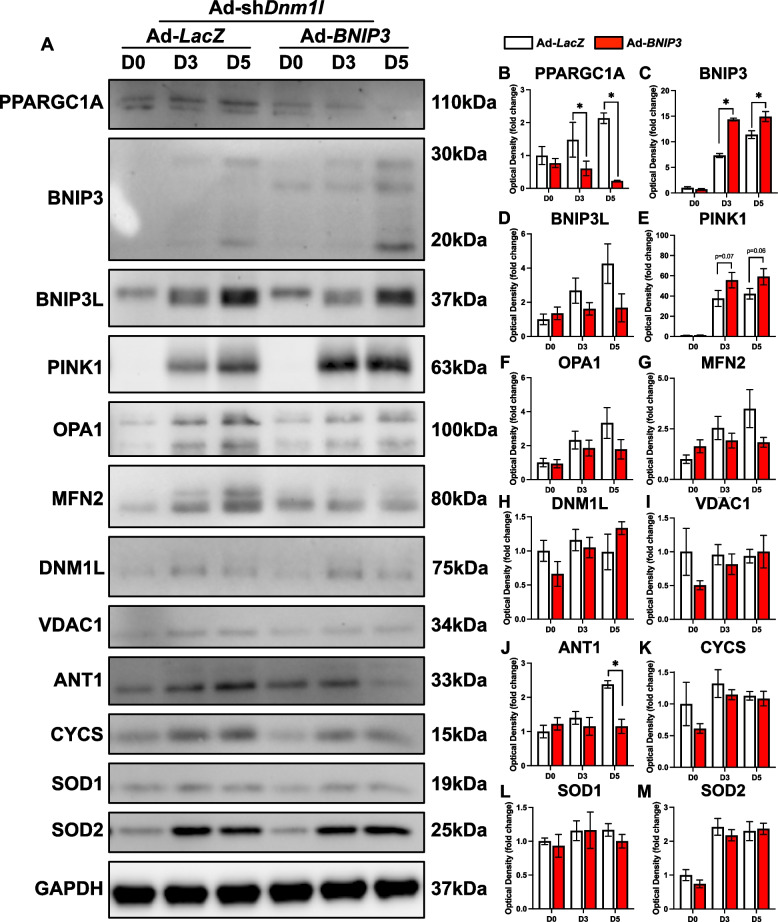
Molecular changes in mitochondrial remodeling proteins in response to BNIP3 OE in fission-deficient cells. Representative immunoblots (**A**) and quantification of (**B**) PPARGC1A, (**C**) BNIP3, (**D**) BNIP3L, (**E**) PINK1, (**F**) OPA1, (**G**) MFN2, (**H**) DNM1L, (**I**) VDAC1, (**J**) ANT1, (**K**) CYCS, (**L**) SOD1, and (**M**) SOD2. * *p* < 0.05 compared to Ad-*LacZ* group within the same time point. *n* = 4 per group

BNIP3 OE in fission-deficient cells did not affect myotube size, differentiation, or fusion indices (Fig. 8A-D). Additionally, BNIP3 OE did not significantly alter parameters of bulk autophagy, as evidenced by unchanged SQSTM1 and LC3B (Fig. 8E-I). These data indicate that the role of BNIP3 on progressing myogenesis and signaling general autophagic pathways is limited in the absence of mitochondrial fission. Additionally, BNIP3 OE decreased PPARGC1A at the mid/late stages of differentiation (−60% at D3, −90% at D5; *p* < 0.05; Fig. 9A-B), while simultaneously increasing PINK1 but not to a significant degree (Fig. 9A, E). Aside from ANT1 (−50% at D5; *p* < 0.05), BNIP3 OE in fission-deficient cells did not alter other markers of mitochondrial dynamics (OPA1 and MFN2) or mitochondrial proteins (Fig. 9A, F-M). This highlights that while BNIP3 can enhance mitophagy in fission-deficient cells, its overexpression alone is not sufficient to compensate for the deficiencies in mitochondrial fission, nor to broadly impact myogenesis in these cells.

### PPARGC1A OE exacerbated myogenic defects in fission-deficient cells

Myogenesis is accompanied by significant mitochondrial remodeling, the result of which is a mitochondrial network that is adept to provide energy at a high enough level to meet the demands of the newly formed myotube [3, 17]. As a result, we questioned whether overexpression of the master regulator of mitochondrial biogenesis PPARGC1A could overcome mitochondrial fission and mitophagy deficiencies to aid in maturation of the mitochondrial network to meet the increased energetic demand of myogenesis.

**Fig. 10:**
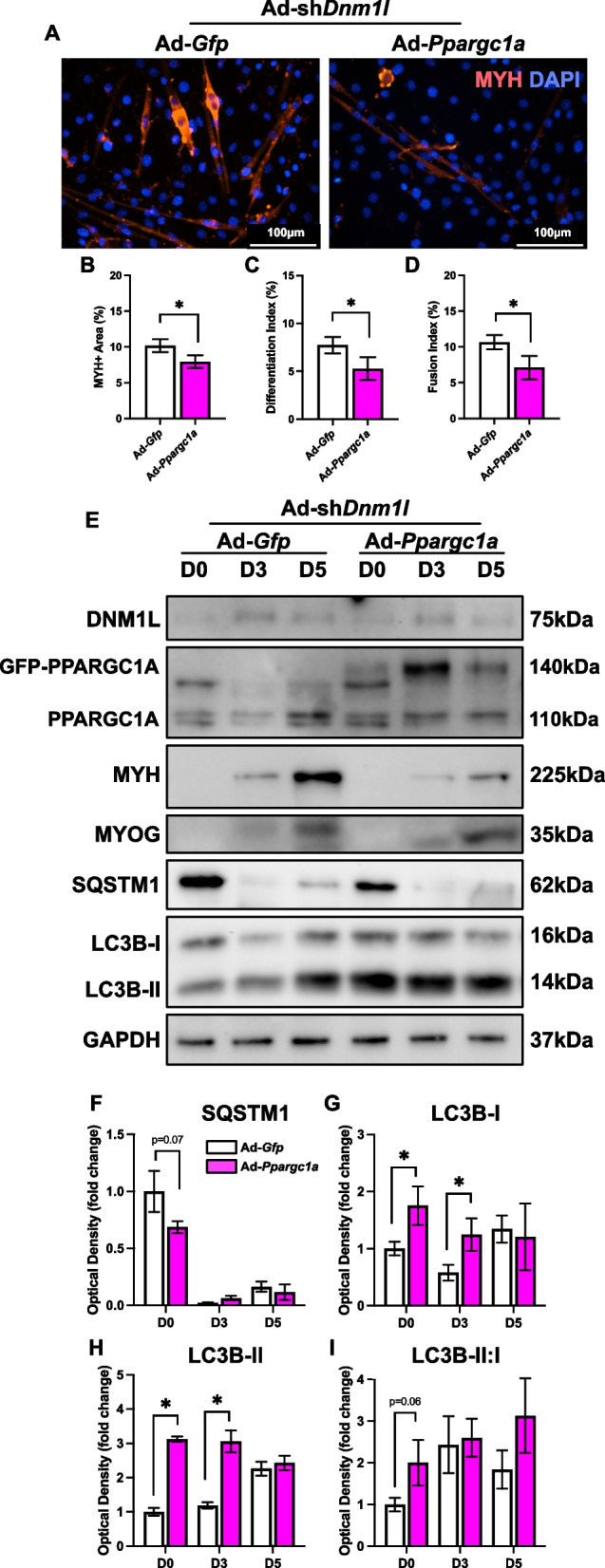
PPARGC1A OE in fission deficient cells exacerbated myogenic defects associated with fission-deficiency. **A** Representative MYH (red) and DAPI (blue) stain of D5 myotubes. Scale bar indicates 100 μm. Quantification of (**B**) MYH + area, (**C**) differentiation index, and (**D**) fusion index. Representative immunoblots (**E**) and quantification of (**F**) SQSTM1, (**G**) LC3B-I, (**H**) LC3B-II, and (**I**) LC3B-II:I. * *p* < 0.05 compared to Ad-*Gfp* group within the same time point. *n* = 4–9 per group

To our surprise, PPARGC1A OE in fission-deficient cells significantly exacerbated myogenic defects, leading to reduced myotube size (−20%; *p* < 0.05), differentiation (−33%; *p* < 0.05), and fusion indices (−34%; *p* < 0.05; Fig. 10A-D). This was accompanied by initial elevations in LC3B-I (+ 75% at D0, + 117% at D3; *p* < 0.05) and LC3B-II (210% at D0, 150% at D3; *p* < 0.05); however, SQSTM1 was only reduced at D0 (*p* = 0.07), and the LC3B-II:I ratio was elevated only at D0 (*p* = 0.06; Fig. 10E-I). This indicates that PPARGC1A OE disrupts myogenesis when fission is impaired, highlighting the need for sequential activation of mitochondrial remodeling processes during myogenesis. The results may also suggest a temporary autophagic response that is not sustained over time in fission-deficient cells.

**Fig. 11:**
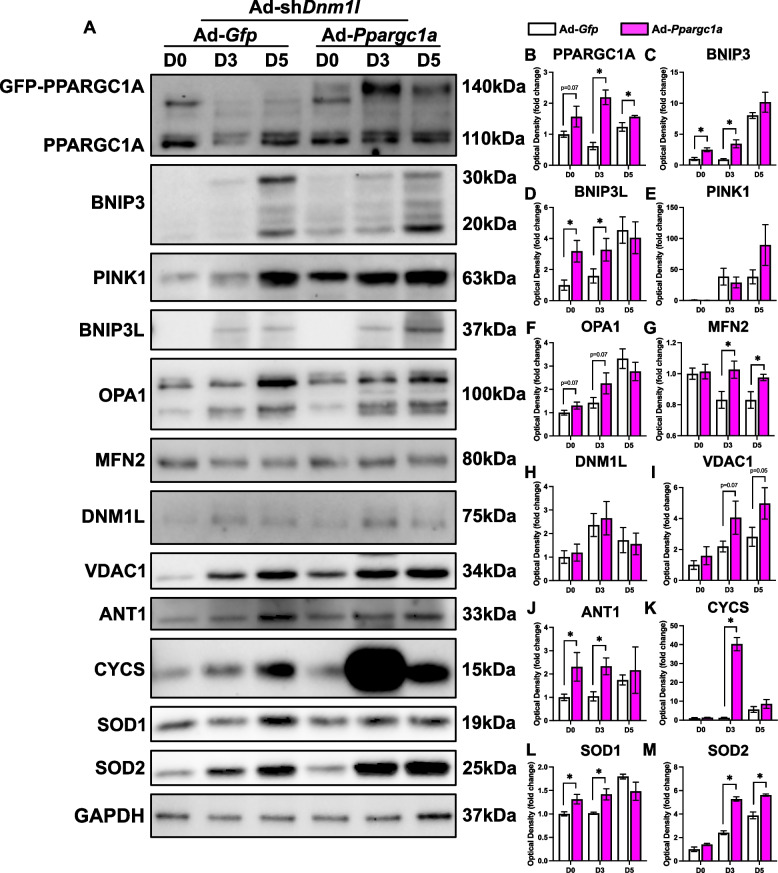
Molecular changes in mitochondrial remodeling proteins in response to PPARGC1A OE in fission-deficient cells. Representative immunoblots (**A**) and quantification of (**B**) PPARGC1A, (**C**) BNIP3, (**D**) BNIP3L, (**E**) PINK1, (**F**) OPA1, (**G**) MFN2, (**H**) DNM1L, (**I**) VDAC1, (**J**) ANT1, (**K**) CYCS, (**L**) SOD1, and (**M**) SOD2. * *p* < 0.05 compared to Ad-*Gfp* group within the same time point. *n* = 4 per group

PPARGC1A OE in fission-deficient cells increased the mitophagy receptors BNIP3 (+ 148% at D0, + 278% at D3; *p* < 0.05) and BNIP3L (+ 227% at D0, 101% at D3; *p* < 0.05; Fig. 11A-D). In addition, PPARGC1A OE elevated the mitochondrial dynamic proteins OPA1 and MFN2, as well as increased the mitochondria-related and antioxidant defence-related proteins including VDAC1, ANT1, CYCS, SOD1 and SOD2 in fission-deficient cells (Fig. 11A, F-M). The elevated mitophagy receptor levels may suggest the cells are attempting to remove damaged mitochondria more efficiently. Alternatively, increased levels of mitophagy receptors may be a consequence of a larger mitochondrial pool and could simply be a by-product of mitochondrial biogenesis.

### Enhanced mitophagic flux and fission are insufficient to recover myogenesis in *Bnip3*^−/−^ cells

**Fig. 12:**
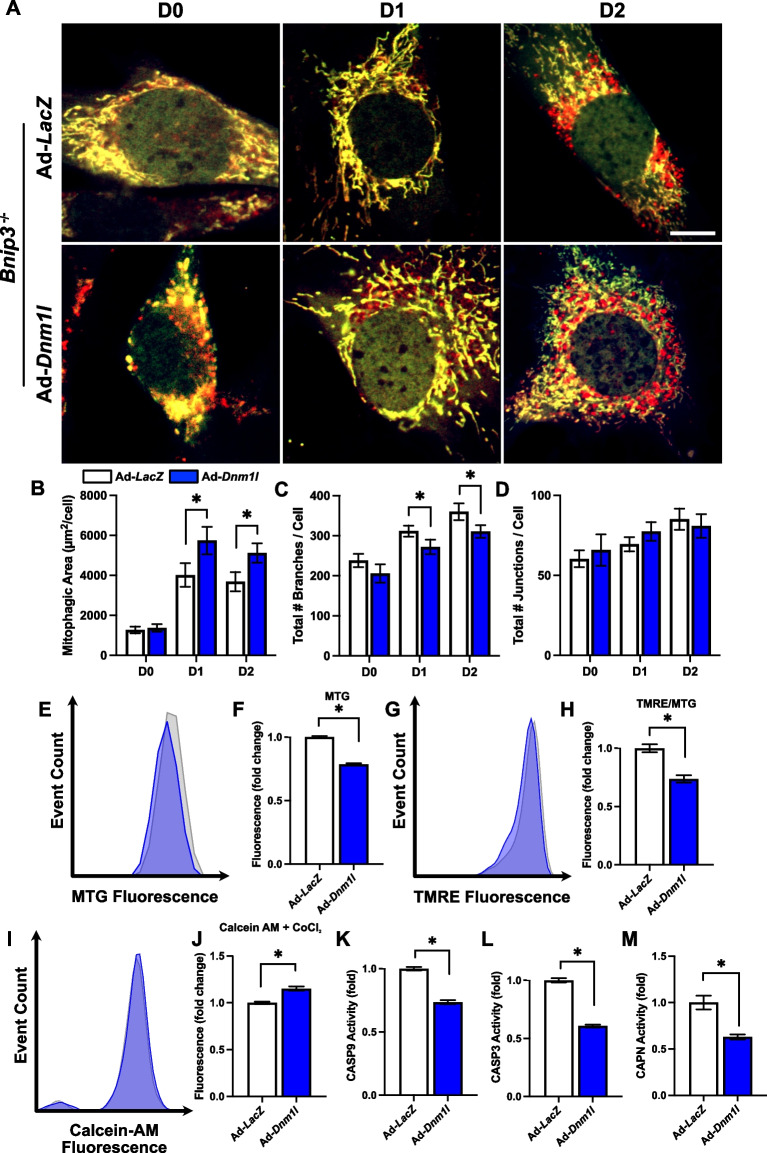
DNM1L OE in *Bnip3*^−/−^ cells increased mitochondrial fragmentation and mitophagy. **A** Representative images of myoblasts transduced with Ad-CMV-*Cox8*-*EGFP*-*mCherry* to detect mitophagic flux. Mitochondria are dual‐labeled EGFP and mCherry (i.e., yellow). EGFP fluorescence is reduced during mitochondria degradation resulting in red fluorescence only (i.e., red puncta) and is indicative of mitophagic flux. Scale bar indicates 10 μm. Quantification of (**B**) mitophagic area, (**C**) total mitochondrial branch count, and (**D**) total mitochondrial junction count per cell. Representative histograms and quantification of (**E–F**) MitoTracker Green (MTG) fluorescence, (**G-H**) TMRE fluorescence, and (**I-J**) Calcein-AM + CoCl_2_ fluorescence. Quantification of (**K**) CASP9, (**L**) CASP3, and (**M**) CAPN activity at D1. * *p* < 0.05 compared to Ad-*LacZ* group within the same time point. *n* = 4–30 per group

To further understand the complex interaction between mitochondrial remodeling processes, we questioned whether enhanced mitochondrial fission could improve mitochondrial remodeling and recover myogenesis independent of BNIP3-mediated mitophagy. We found that DNM1L OE in *Bnip3*^*−/−*^ cells reduced the total number of mitochondrial branches (−13% at D1 and 14% at D2; *p* < 0.05) and was accompanied by increased mitophagic flux in differentiating myoblasts at D1 (+ 42%; *p* < 0.05) and D2 (+ 41%; *p* < 0.05; Fig. 12A-D). This coincided with our earlier results demonstrating enhanced mitophagic flux in DNM1L OE cells (Fig. 1); however, this is unique because enhanced mitophagic flux was independent of BNIP3; thus, suggesting alternative mitophagy mediated mechanisms that are active in these cells during an enhanced fission state. Furthermore, DNM1L OE in *Bnip3*^*−/−*^ cells decreased MTG fluorescence (−22%; *p* < 0.05), TMRE fluorescence (−27%; *p* < 0.05), and the activity of CASP3 (−40%; *p* < 0.05), CASP9 (−27%; *p* < 0.05), and CAPN (−38%; *p* < 0.05), while also reducing mitochondrial MPTP opening at D1 (+ 15%; *p* < 0.05); Fig. 12E-M). These findings collectively indicate a role of DNM1L in modulating mitochondrial function and downstream mitochondrial apoptotic signaling during myogenic differentiation independent of BNIP3-mediated mitophagy.

**Fig. 13:**
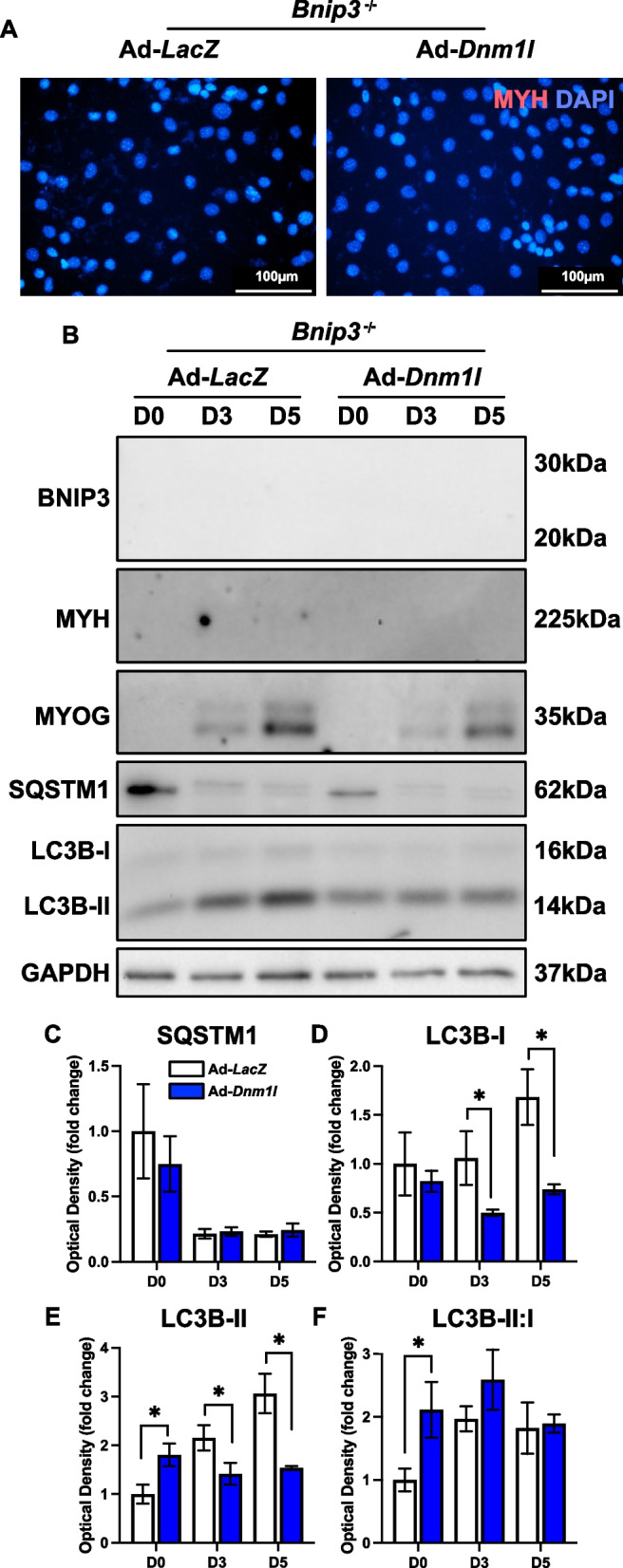
DNM1L OE in *Bnip3*^−/−^ cells does not modify myogenic differentiation. **A** Representative MYH (red) and DAPI (blue) stain of D5 cells. Scale bar indicates 100 μm. Representative immunoblots (**B**) and quantification of SQSTM1, (**D**) LC3B-I, (**E**) LC3B-II, and (**F**) LC3B-II:I. * *p* < 0.05 compared to Ad-*LacZ* group within the same time point. *n* = 4 per group

**Fig. 14:**
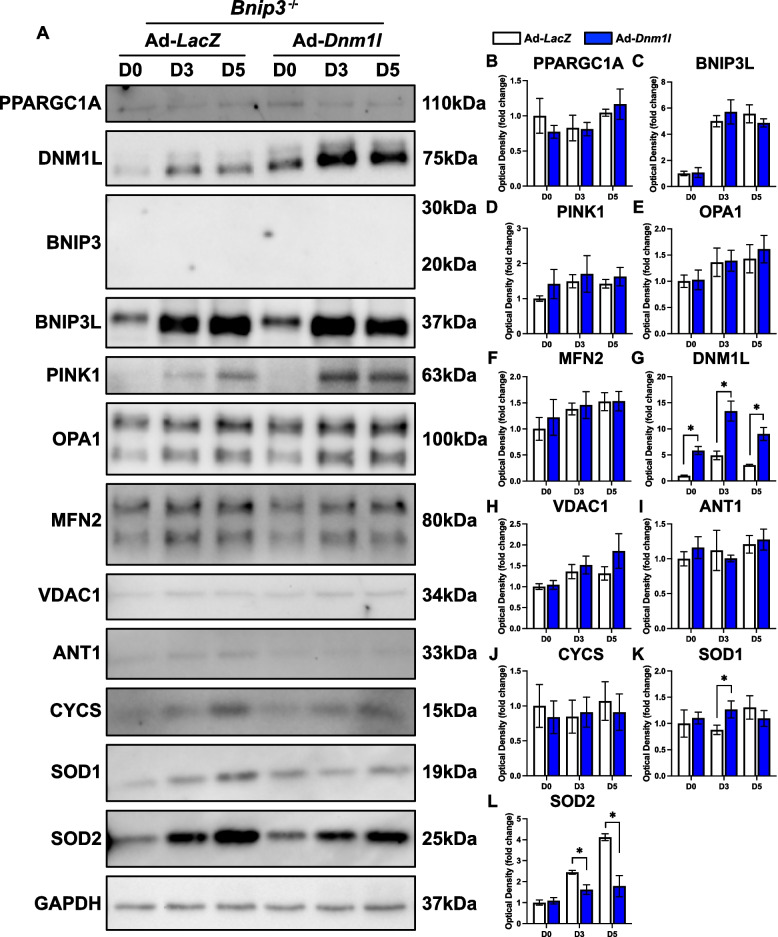
Molecular changes in mitochondrial remodeling proteins in response to DNM1L OE in *Bnip3*^−/−^ cells. Representative immunoblots (**A**) and quantification of (**B**) PPARGC1A, (**C**) BNIP3L, (**D**) PINK1, (**E**) OPA1, (F) MFN2, (**G**) DNM1L, (**H**) VDAC1, (**I**) ANT1, (**J**) CYCS, (**K**) SOD1, and (**L**) SOD2. * *p* < 0.05 compared to Ad-*LacZ* group within the same time point. *n* = 4 per group

However, DNM1L OE in *Bnip3*^*−/−*^ cells did not recover the defects observed in myotube size, differentiation, or fusion indices (Fig. 13A). This suggests that BNIP3 plays a crucial role in myogenesis, with its influence possibly extending beyond its known function in mitophagy. Nonetheless, we show that DNM1L OE in *Bnip3*^*−/−*^ cells reduced LC3B-I (−50% at D3 and −47% at D5; *p* < 0.05) and altered LC3B-II (+ 80% at D0, −35% at D3 and −50% at D5; *p* < 0.05) during differentiation, indicating rapid changes in autophagosome formation (Fig. 13B-F). Interestingly, DNM1L OE in *Bnip3*^*−/−*^ cells did not alter key proteins related to mitochondrial biogenesis (i.e., PPARGC1A), mitophagy (i.e., BNIP3L, PINK1), mitochondrial dynamics (i.e., OPA1, MFN2), or other mitochondrial-related functions (i.e., VDAC1, ANT1, and CYCS), but did alter the antioxidant defence proteins SOD1 (+ 44% at D3; *p* < 0.05) and SOD2 (−35% at D3 and −57% at D5; *p* < 0.05; Fig. 14A-L). This suggests that DNM1L may specifically affect mitochondrial quality control mechanisms rather than broader aspects of mitochondrial remodeling including mitochondrial biogenesis in *Bnip3*^*−/−*^ cells.

## Discussion

Robust cellular remodeling is a fundamental aspect that underpins myogenesis. A critical component of this remodeling process is mitochondrial remodeling, which is crucial in ensuring the energy demands of newly formed myotubes are met to fully support its physiological functions. Despite the recognized importance of mitochondrial remodeling in myogenesis [3, 17], the precise mechanisms and interactions within this process remain inadequately understood. In the present study, we provide evidence that mitochondrial remodeling processes must occur in a specific, sequential order for effective myogenesis. Our findings indicate that: 1) alterations in mitochondrial fission regulates mitophagy and the progression of myogenesis, 2) enhanced mitophagy or mitochondrial biogenesis alone are unable to restore myogenic defects associated with fission-deficiency, underscoring the necessity of proper mitochondrial fission during myogenesis, and 3) enhanced mitochondrial fission in BNIP3-deficient cells fails to rescue differentiation. These data highlight the intricate balance and coordination of mitochondrial dynamics processes for successful skeletal muscle cell differentiation, providing new insights into the cellular mechanisms underlying myogenesis.

### Alterations in mitochondrial fission modulate mitophagy and progression of myogenesis

Mitochondrial fission is a critical process that ensures proper mitochondrial distribution and function within cells, playing a vital role in maintaining cellular energy balance and homeostasis. This dynamic remodeling of mitochondria facilitates the removal of damaged mitochondria through mitophagy and supports the generation of higher functioning mitochondria [3, 5, 17–20]. Alterations in mitochondrial dynamics can significantly affect the differentiation of various cell types by disrupting mitochondrial function and energy production. In certain cell types, a shift towards mitochondrial fusion is essential for efficient differentiation, such as in induced pluripotent stem cells and immune cells [21, 22]. In this context, the fusion of the mitochondrial network is necessary to reprogram the cells and enhance oxidative phosphorylation [21, 22]. A similar form of metabolic reprogramming is observed in muscle cells during differentiation. However, there appears to be a greater need for mitochondrial fission, especially during the early stages of the differentiation program to support the metabolic reprogramming of these cells [23]. The need for mitochondrial fission is also corroborated by more recent work in skeletal muscle stem cells (i.e., satellite cells) that lack DNM1L [24]. This study found that in vivo regeneration is accompanied by a temporal increase in DNM1L to aid in satellite cell proliferation via activation of mitophagy [24]. Conversely, the loss of DNM1L-mediated fission in satellite cells significantly limits mitophagy and activation of satellite cells; thereby, leading to an impaired regenerative response [24]. Taken together, mitochondrial dynamics is critical in cellular differentiation.

There is also evidence that impaired mitochondrial fission is detrimental to myogenic differentiation [11, 15]; however, there is less direct evidence linking the role of mitochondrial hyper-fragmentation during myogenesis. Previous studies have demonstrated that DNM1L OE impaired muscle development in young mice by preventing the maturation of the mitochondrial network [25, 26]. Our data indicate a similar pattern such that DNM1L OE cells exhibit reduced myotube size, which appears to be linked to the increased fragmentation of the mitochondrial network, particularly during the early phase of the differentiation program; a timepoint of enhanced mitophagic flux. Although previous studies suggest that mitochondrial fission is a preceding step to cell death [27], our findings indicate that DNM1L OE reduced CASP9 activity; albeit while increasing MPTP opening. The reasons behind this paradox are not fully understood. One possibility is the persistence of “dysfunctional” mitochondria that have not been effectively targeted for mitophagy. MPTP opening may be sensitive to changes in the overall mitochondrial network morphology. In support of this, inhibition of mitochondrial fission prevents MPTP opening, whereas ablation of MFN2 increases mitochondrial fragmentation and heightens the susceptibility to MPTP opening in cardiomyocytes [28, 29]. Alternatively, the greater degree of mitophagic flux may be limiting the activation of the mitochondrial caspase, CASP9. Several studies have demonstrated the importance of CASP9 activation during myogenesis. For instance, genetic knockdown of *Casp9*, overexpression of anti-apoptotic molecules such as BCL2 like 1 (BCL2L1), or chemical inhibition of apoptotic peptidase activating factor 1 (APAF1) all result in reduced myoblast fusion, impaired myotube formation, and a lack of CASP3 activation [30, 31]. Conversely, our data suggests that overactive CASP9 can impede differentiation and trigger apoptosis, an effect that can be mitigated by modulating CASP9 activity through transduction with dominant-negative CASP9 [4].

Previous studies have demonstrated a link between mitochondrial fission, myogenesis, and apoptosis [11, 15]. More specifically, increasing concentrations of mdivi-1 and expression of a dominant negative DNM1L^K38A^ reduce MYOG and myosin formation in C2C12 and primary myoblasts, and cause classical features of apoptosis including CASP3 activation and TUNEL + nuclei [11, 15]. In agreement with these findings, we show impaired differentiation, myoblast fusion, and overall myotube size in DNM1L KD cells, and augmented apoptotic signaling through CASP3. We add to this literature by now demonstrating that these defects are coupled with a hyperfused mitochondrial network and suppressed mitophagic flux, and mitochondrial apoptotic signaling via CASP9 activation. Interestingly, DNM1L KD reduced PPARGC1A, BNIP3, MFN2, and SOD2. This suggests that the lack of mitochondrial fission can signal, likely indirectly, several other mitochondrial remodeling processes. Collectively, these data indicate that mitochondrial fission is crucial not only for the progression of myogenesis but also for maintaining mitochondrial death signaling processes by activating mitophagy.

### Overexpressing molecules involved in mitophagy or mitochondrial biogenesis are insufficient to rescue myogenic defects due to fission-deficiency

In the present study, we have established the necessity of mitochondrial fission for myogenesis. We then aimed to determine if modifying other mitochondrial remodeling processes, particularly mitophagy and mitochondrial biogenesis, could improve differentiation in DNM1L-independent mechanisms. Previous studies on primary muscle stem cells from geriatric human patients and aged mice have revealed a reduction in autophagic and mitophagic flux, which is associated with a decline in the activation and proliferation of the stem cell pool, ultimately leading to impaired regenerative capacity [32]. Notably, treatment with the autophagy-inducing agent rapamycin in aged mice and geriatric human muscle stem cells improved mitophagic flux and reversed the accumulation of dysfunctional mitochondria [32]. Therefore, it stands to reason that restoration of mitophagy in a fission-deficient state may restore myogenesis. Given the importance of BNIP3 in mitophagy and myogenesis [4], we overexpressed BNIP3 in fission-deficient cells. Although BNIP3 overexpression enhanced mitophagic flux, it failed to recover myogenic defects in fission-deficient cells. Interestingly, BNIP3 OE in these cells also led to decreased CASP3 and CASP9 activity. This reduction in CASP9 and CASP3 activation could be problematic, as the activation of these enzymes is essential for myogenesis. As previously mentioned, CASP9 activation is required for myogenesis; however, a balance is required which ultimately influences the differentiation-apoptosis axis. Therefore, BNIP3 OE and the resulting increase in mitophagic flux in fission-deficient cells may lead to excessive mitochondrial degradation, thereby impairing the downstream activation of CASP9 and CASP3, which is crucial for myogenesis.

To date, no studies have specifically investigated PPARGC1A overexpression in fission-deficient cells. Nevertheless, previous work has established a relationship between autophagy and PPARGC1A during myogenic differentiation [4, 5]. For example, enhanced nuclear localization of PPARGC1A occurs during the later stages of myogenesis, while inhibition of autophagy by bafilomycin A restricts nuclear translocation of PPARGC1A [5]. Further, PPARGC1A is reduced in *Atg7* knockdown cells and *Bnip3*^−/−^ cells [4]. Moreover, mitochondrial biogenesis appears to facilitate the renewal of the mitochondrial network following mitophagy, thereby producing more functionally efficient mitochondria that meet the energetic demands of myotubes [5]. Consistent with the literature, we found reduced PPARGC1A in fission-deficient cells. However, to our surprise, PPARGC1A overexpression in fission-deficient cells significantly hindered myogenic differentiation, and was associated with increased levels of autophagosome proteins, mitophagy receptors, mitochondrial dynamics proteins, and other mitochondrial-related molecules.

### BNIP3 governs myogenic differentiation beyond its role in mitophagy

Based on the data presented, it appears that efficient mitochondrial fission and mitophagy are essential for myogenesis. In the absence of mitochondrial fission, significant defects in mitophagy occur, leading to impaired myogenesis. Although prior studies have established that fission is necessary for BNIP3-mediated mitophagy [16]; to our knowledge, the effect of DNM1L OE on enhancing mitophagy independently of BNIP3 has not been examined. Interestingly, we observed that *Bnip3*^−/−^ cells continued to show mitophagic activity but failed to differentiate into myotubes. This finding aligns partially with earlier findings from our group, which noted differentiation defects in *Bnip3*^−/−^ cells; however, changes in mitophagic flux in these cells was not explored [4]. In the present study, DNM1L OE led to increased mitochondrial network fragmentation (i.e., reduced branching) and enhanced mitophagic flux, yet it did not restore myogenic differentiation in these cells. This observation suggests a potential requirement for BNIP3 in regulating myogenic differentiation, possibly through its roles in mitophagy or the modulation of other signaling cascades. Interestingly, the persistence of mitophagic flux in *Bnip3*^−/−^ cells indicates the activation of alternative mitophagy pathways. Moreover, this observation aligns with existing knowledge that multiple redundant mitophagy pathways exist, which can compensate for the loss of a single molecule [33, 34]. This redundancy highlights the complexity of studying mitophagy mechanisms.

### Limitations, future direction, and translational relevance

In the present study, we demonstrated that mitochondrial fission is essential for myogenic differentiation and that activating other remodeling processes is insufficient to compensate for fission deficiency and its associated myogenic defects. While these findings provide valuable mechanistic insights into the crosstalk between mitochondrial remodeling processes, there are some limitations. Notably, these experiments were conducted in immortalized murine myoblasts, making it unclear whether these mechanisms play a similar governing role in other cell types, such as rodent or human primary myoblasts. Future research should investigate these mechanisms in primary myoblasts, particularly focusing on specific cellular states, including quiescence, activation, and differentiation. Additionally, we observed that increasing mitochondrial fission enhanced mitophagic flux in *Bnip3*^−/−^ cells; however, this did not restore their differentiation capacity. Future research should explore alternative proteins that may regulate mitophagy in the absence of BNIP3. Potential candidates include mitophagy-related molecules such as FUN14 domain containing 1 (FUNDC1), BCL2 like 13 (BCL2L13), BNIP3L, or certain RAB-related proteins, which may control mitophagic flux independently of BNIP3.

These findings hold significant therapeutic potential, suggesting several important strategies. For instance, 1) prioritizing the "upstream" remodeling process, such as mitochondrial fission, before targeting mitophagy to ensure proper mitochondrial quality control; 2) recognizing that mitophagy can occur independently of specific mitophagy molecules, highlighting the need to strategically select the most effective targets for therapeutic intervention; and 3) understanding that some mitophagy molecules not only regulate mitophagy but also influence cellular pathways/functions such as differentiation and apoptosis, thus emphasizing the need to consider the broader consequences of targeting mitophagy.

## Conclusion

This study explored the complex interplay between mitochondrial remodeling processes and myogenesis, revealing that the orchestrated regulation of mitochondrial fission, mitophagy, and biogenesis is fundamental to efficient skeletal muscle cell differentiation. We show that mitochondrial fission acts as a pivotal gateway that facilitates subsequent cellular processes such as mitophagy and mitochondrial biogenesis. We demonstrate that genetic alterations in the expression of key mitochondrial remodeling proteins involved in mitophagy and mitochondrial biogenesis are insufficient on their own to address defects in myogenesis due to mitochondrial fission-deficiency. This suggests that a lack of proper fission disrupts mitochondrial remodeling processes, specifically mitophagy, which is needed for the progression of myogenesis. Insights from this study highlight a potential therapeutic angle, emphasizing the need for strategies that can restore or enhance the natural sequence of mitochondrial remodeling, ensuring that each phase contributes positively to myogenesis. These strategies could be particularly beneficial in treating muscular diseases where mitochondrial dysfunction is a contributing factor, offering a route to enhance muscle repair and functional recovery through targeted modulation of mitochondrial dynamics.

## Data Availability

The datasets during and/or analysed during the current study available from the corresponding author on reasonable request.

## Abbreviations

Ad: Adenovirus
ANT1: Adenosine nucleotide translocator 1
APAF1: Apoptotic peptidase activating factor 1
ATG7: Autophagy related 7
BCL2L1: BCL2 like 1
BCL2L13: BCL2 like 13
BNIP3: BNIP3
BNIP3L: BNIP3 like
CAPN: Calpain
CASP: Caspase
CYCS: Cytochrome c
DNM1L: Dynamin 1 like
FUNDC1: FUN14 domain containing 1
GFP: Green fluorescence protein
KD: Knockdown
MAP1LC3B/LC3B: Microtubule associated protein 1 light chain 3 beta
MFN2: Mitofusin 2
MPTP: Mitochondrial permeability transition pore
MTG: Mitotracker green
MYH: Myosin
MYOG: Myogenin
OE: Overexpression
OPA1: Optic atrophy 1
PINK1: PTEN induced kinase 1
PPARGC1A: PPARG coactivating 1 alpha
PRKN: Parkin RBR E3 ubiquitin protein ligase
RAB: RAB, member RAS oncogene family
SOD: Superoxidase dismutase
SQSTM1: Sequestosome 1
TMRE: Tetramethylrhodamine ethyl ester
VDAC1: Voltage dependent anion channel 1
